# Precision-cut lung slices as an *ex vivo* model to study *Pneumocystis murina* survival and antimicrobial susceptibility

**DOI:** 10.1128/mbio.01464-23

**Published:** 2023-12-20

**Authors:** Ferris T. Munyonho, Robert D. E. Clark, Dong Lin, Mst Shamima Khatun, Dora Pungan, Guixiang Dai, Jay K. Kolls

**Affiliations:** 1 Center for Translational Research in Infection and Inflammation Tulane School of Medicine, New Orleans, Louisiana, USA; Duke University, Durham, North Carolina, USA

**Keywords:** *Pneumocystis *pneumonia, *Pneumocystis murina*, PCLS

## Abstract

*Pneumocystis* pneumonia (PCP) is a serious fungal lung infection caused by *Pneumocystis jirovecii* in immunosuppressed individuals. The lack of viable *in vitro/ex vivo* PCP models has greatly hindered the progress in studying the biology of these fungi, the host/pathogen interactions, as well as antifungal susceptibility testing. In this study, we show the utility of precision-cut lung slices (PCLS) to support the survival of *Pneumocystis murina in vitro*. We cultured PCLS tissue derived from wild type and immunocompromised mice with a *P. murina* inoculum in submerged or air-liquid interface models for up to 14 days. We isolated total RNA from the cultured lung tissues at days 0, 3, 7, 10, and 14 and analyzed for the expression of host lung genes and *P. murina* genes (*Gsc1* for asci and *Sp* for trophs) by real-time quantitative PCR. When cultured in media alone, *P. murina* died gradually within a few days. However, when cultured on PCLS, both the troph and ascus forms survived throughout the incubation period of 2 weeks. Moreover, immunohistochemistry staining of *P. murina* inoculated PCLS sections using polyclonal anti-*Pneumocystis* sera and showed evidence of fungal aggregation and possible biofilm formation. Additionally, *in vitro* (PCLS) antibiotic susceptibility testing using commonly used antifungal drugs confirmed successful targeting of the troph and ascus forms by trimethoprim sulfamethoxazole and the ascus form by echinocandins.

**IMPORTANCE:**

Our study reveals the potential of precision-cut lung slices as an *ex vivo* platform to study the growth/survival of *Pneumocystis* spp. that can facilitate the development of new anti-fungal drugs.

## INTRODUCTION

*Pneumocystis* pneumonia (PCP) is a life-threatening lung infection caused by an opportunistic fungal pathogen, *Pneumocystis jirovecii*, in immunosuppressed individuals (1–3). *P. jirovecii* remains a clinically potent human fungal pathogen globally and has been recently listed among the top 19 fungal priority pathogens by the World Health Organization (4). There are few treatment options for PCP (5, 6), and morbidity as well as mortality due to this infection remain high and poorly controlled, especially in developing countries. In previous years, PCP cases have been largely attributed to HIV/AIDS. However, recent studies have shown that other factors leading to immunosuppression, including malignancies, inherited immunodeficiency, and subjection to immunosuppressive therapies, have become the most prevalent risk factors for PCP infections and hospitalizations (1, 7–9). The expansion in susceptible populations as well as the limitations in treatment options suggest an urgent need for new treatment strategies for PCP infection (7).

The search for new antifungal therapies and therapeutic targets for PCP has been significantly hindered by the lack of a continuous *in vitro*/*ex vivo* culture system for *Pneumocystis* species. Moreover, the failure to culture *Pneumocystis* spp. outside the mammalian host lung has hampered progress in studying the biology of these fungal pathogens as well as their mechanism of attachment to the host lung cells (10). Despite the efforts, several attempts to propagate *Pneumocystis* spp. *in vitro/ex vivo* have not been successful (7, 11). Cushion and colleagues reviewed the numerous attempts made by different researchers to propagate *Pneumocystis* spp. *in vitro* but with limited success (7). Hence, there is a critical need to develop an *in vitro*/*ex vivo* system that supports the growth/survival of these fungi to allow antifungal susceptibility testing as well as the determination of host-pathogen interactions.

Precision-cut lung slices (PCLS), uniformly sliced human or animal lung tissues generated by a vibrating microtome, have recently emerged as a novel *ex vivo* organotypic system for modeling lung inflammatory diseases (12, 13). Several studies have reported the successful utilization of PCLS as an *ex vivo* platform to model various lung disorders such as asthma, chronic obstructive pulmonary disease, and idiopathic pulmonary fibrosis (14–16). PCLSs have the main advantage of retaining a phenomenal cellular complexity and lung architecture, which provides a platform to study respiratory pathogens in an environment that closely mimic the *in vivo* lung conditions (12). Moreover, the PCLS model allows the generation of large numbers of uniformly cut tissue sections from a single lung tissue enabling multiple variables to be tested simultaneously. This reduces the number of animals and their discomfort as well as the time needed for infection studies (12, 15). Furthermore, PCLSs are reproducible and allow for high-resolution imaging of the lung tissue and cellular functions in three dimensions (17, 18). Herein, we report the use of PCLS as a potential *ex vivo* platform to study *Pneumocystis murina* survival/growth.

## RESULTS

### Naïve PCLS culture in submerged well and air-liquid interface models

**Fig 1:**
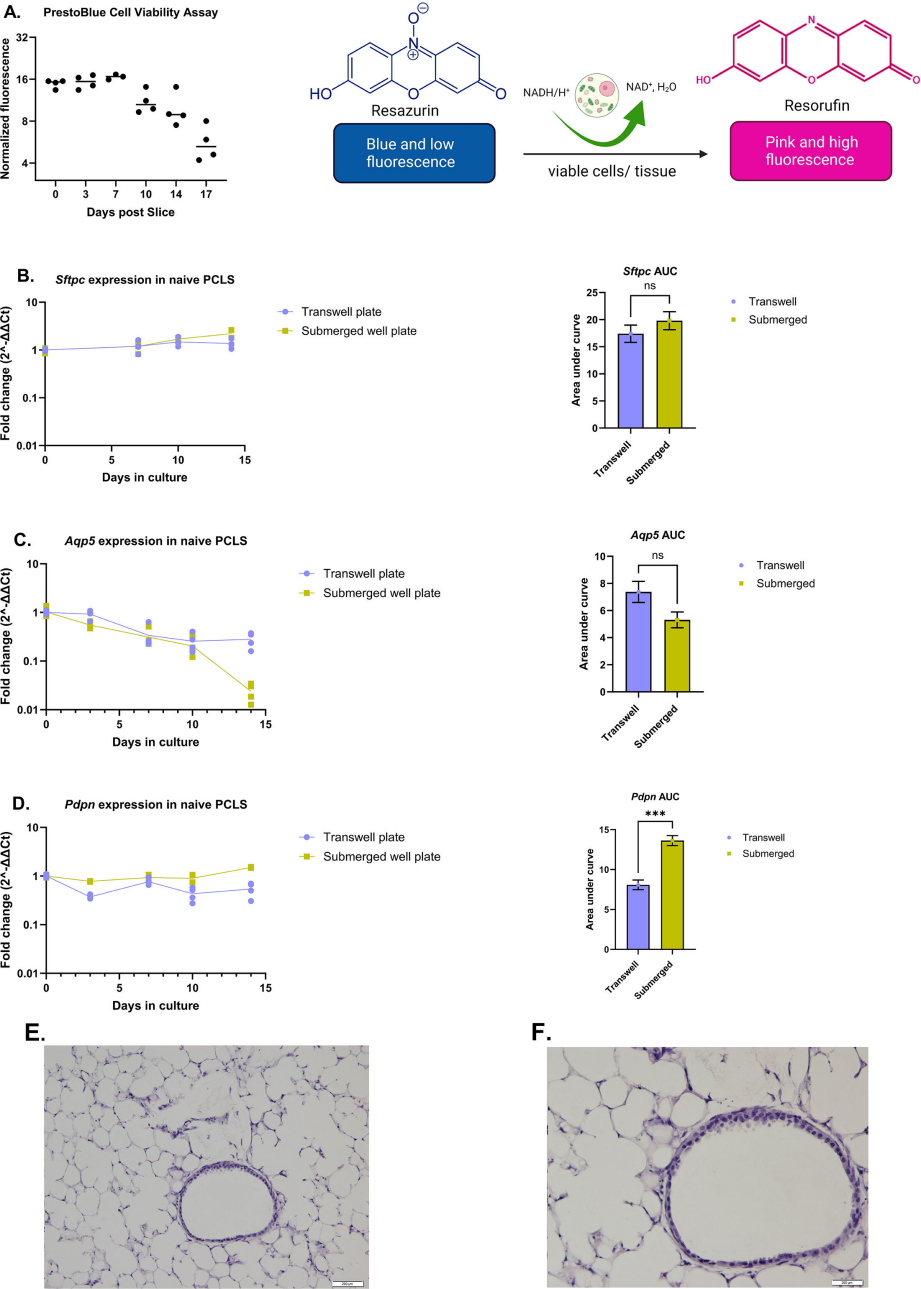
Viability assays for the cultured naïve PCLS and expression of alveolar type I (*Aqp5* and *Pdpn*) and type II (*Sftpc*) cell markers in submerged and air-liquid interface PCLS models over time. *Hprt* was used as a housekeeping gene to normalize lung gene expressions. (**A** left panel) PrestoBlue cell viability assays for cultured naïve PCLS over time. Fluorescence was measured at 560/590 nm (excitation/emission). (**A** right panel) Illustration of the reaction basis of PrestoBlue cell viability assay, created with BioRender.com. (**B–D**) *Sftpc*, *Aqp5*, and *Pdpn* expression profiles in naïve PCLS during a 14-day culture period and the respective area under curve (AUC). (**E**) 10× magnification and (**F**) 20× representative magnification images of hematoxylin and eosin-stained naïve lung tissue sections after being cultured in DMEM for 14 days. AUC was computed by considering the area under each gene expression profile between day 0 and day 14, using the GraphPad Prism software. Statistical comparisons between groups were done by two-tailed unpaired *t* test, with Welch’s correction, of the AUC of the respective gene expression profiles. For (**A**), each data point represents the average fluorescent intensity for three PCLS and *n* (total number of lung slices/PCLS) = 12 per time point. For (B–D), *n* = 4 lung slices per time point. *P* values were denoted as follows: ns: *P* ˂ 0.05, * : P ≤ 0.05, ** : P ≤ 0.01, *** : P ≤ 0.001, and **** : P ≤ 0.0001.

We first assessed the viability and alveolar architecture of naïve lung slices cultured in Dulbecco’s Modified Eagle Medium (DMEM) over time. Lungs were inflated with warm low-melting agarose and sectioned, upon solidification, on a vibratome at 300 µm thickness. Subsequently, the viability of the lung slices was tested using PrestoBlue cell viability assay, which utilizes the reducing power of live cells to convert resazurin (blue in color) to a high fluorescent molecule, resorufin (pink), as illustrated in Fig. 1A (right panel). A similar assay, using AlamarBlue, was utilized by Bryson and colleagues (19) to assess the viability of chicken-derived PCLS up to 44 days of culture. Previous studies have shown that human or murine PCLS can maintain viability and structural integrity in culture for 15 days (20, 21). We thus assessed the viability of our murine PCLS quantitatively for up to 17 days by measuring the fluorescence intensity of the supernatants obtained after incubating PCLS in a mixture of DMEM and PrestoBlue for 1 hour at 37°C using a microplate reader. The fluorescence intensity of the cultured PCLS was normalized to the negative control (heat-killed PCLS). Although there was a drastic decrease in fluorescence intensity at day 17, the fluorescence intensity stayed above the background detected in the negative control for 17 days in culture (Fig. 1A), suggesting the presence of viable cells in the PCLS over the incubation period.

Studies have shown that *Pneumocystis* spp. reside predominantly in the alveolar space of the host lung (22). Moreover, it has been shown that the binding of the trophs [one of the two predominant life forms of *Pneumocystis* (PC) to alveolar epithelial cells], particularly type I cells, represents a critical component of the organism’s life cycle (23, 24). With that in mind, we assessed the expression of type I and type II alveolar cell markers in the cultured PCLS in either submerged or air-liquid interface models over time, up to 14 days. Subsequently, total RNA was isolated from the cultured PCLS at days 0, 3, 7, 10, and 14 of culture. The expression of podoplanin (*Pdpn*) and aquaporin 5 (*Aqp5*; alveolar type I cell markers); surfactant protein c (*Sftpc*; an alveolar type II cell marker); and a housekeeping gene, hypoxanthine guanine phosphoribosyltransferase (*Hprt*), was assessed by quantitative reverse transcription PCR (RT-qPCR). Consistent with the results from the PrestoBlue cell viability assays (Fig. 1A left panel), the data showed significant expression of the tested lung genes throughout the incubation period (Fig. 1B through D), indicating the presence of viable type I and type II cells in the PCLS over the incubation period. We did note some decline in *Aqp5* in submerged slices suggesting that type I cells may have reduced viability in this condition. However, hematoxylin and eosin (H&E)-stained sections of cultured PCLS (from transwells) confirmed the presence of normal lung alveolar architecture at day 14 of culture (Fig. 1E and F).

### Survival of *P. murina* in submerged well and air-liquid interface PCLS culture models

**Fig 2:**
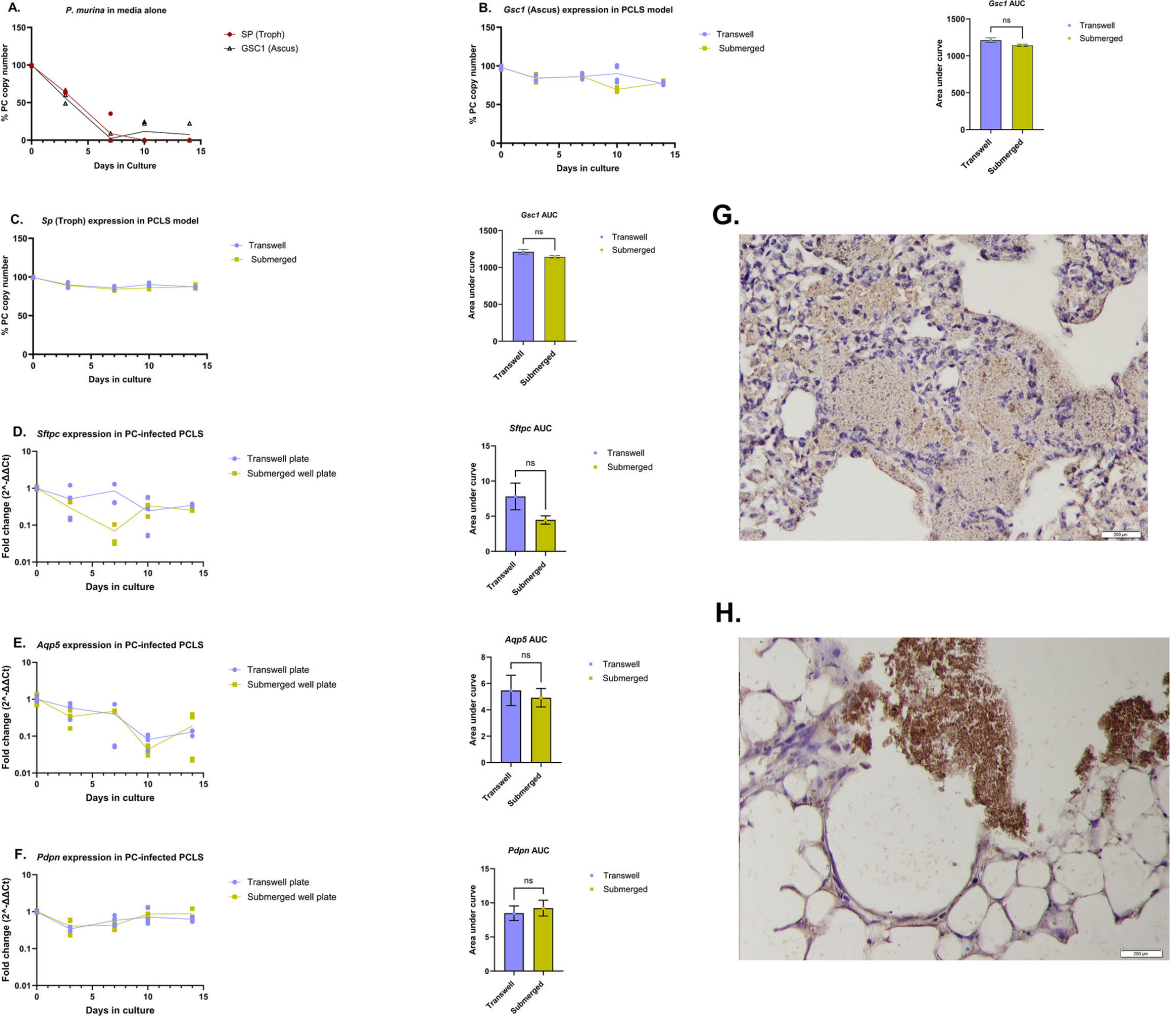
Survival of *P. murina* organisms on *Rag2^-/-^Il2rγ^-/-^* mice-derived PCLS over time. (**A**) PC gene expression profiles for the *P. murina* troph (*Sp*)- and ascus (*Gsc1*)- specific transcripts in DMEM alone, over time. (**B**) Gene expression profile for the ascus (*Gsc1*)- specific transcript over time in submerged and air-liquid interface PCLS models. (**C**) Gene expression profile for the troph (*Sp*)-specific transcript over time in submerged and air-liquid interface PCLS models. (**D–F**) *Sftpc*, *Aqp5*, and *Pdpn* expression in *P. murina* inoculated PCLS models over time and the respective AUC. (**G**) Representative image (20×) of IHC-stained positive control of *in vivo* infected *Rag2^-/-^Il2rγ^-/-^* mouse lung showing brown stained fungi in the alveolar space. (**H**) Representative image (20×) of IHC staining of day 14 *Rag2^-/-^Il2rγ^-/-^*PCLS cultured with *P. murina* showing brown stained aggregates of fungi which is suggestive of fungal biofilms. For **B–F**, AUC was computed as described in Fig. 1. For **A–C**, day 0 denotes the starting inoculum. For **A–F**, *n* = 4 lung slices per time point, and a statistical analysis was done as described in Fig. 1 with *P* denoted as ns: *P* ˂ 0.05, * : P ≤ 0.05, ** : P ≤ 0.01, *** : P ≤ 0.001, and **** : P ≤ 0.0001.

Having established that the PCLS system maintains a normal lung alveolar architecture and viability in culture over time, we hypothesized that it could be a useful *ex vivo* platform to study the biology of PC. To that end, we assessed the survival/growth of *P. murina* on PCLS by monitoring the expression of a troph-specific transcript, serine protease (*Sp*), and an ascus-specific transcript, *Gsc1* (25), over a period of 14 days. The trophozoite-enriched expression marker, *Sp* (PNEG_02319), and the ascus-enriched expression marker, *Gsc1* (PNEG_03180), were previously validated as life-form specific makers for PC that can be utilized to track the state of PC life cycle *in vivo* (25). Since PC thrives in immunocompromised lung environments, we initially studied lungs obtained from male or female immunodeficient, *Rag2^-/-^Il2rγ^-/-^*, mice to avoid any inhibition of fungal growth from lung natural killer (NK) cells or innate lymphoid cells. Equal aliquots (120 µL per well) of *P. murina* inoculum, from the same stock, were cultured in submerged well or air-liquid interface PCLS models for 14 days. Total RNA was harvested at days 0, 3, 7, 10, and 14, followed by the assessment of the expression of fungal (*Sp* and *Gsc1*) and host lung (*Sftpc*, *Aqp5*, *Pdpn*, and *Hprt*) genes by RT-qPCR. *P. murina* cultured in DMEM alone died (as measured by RNA integrity) within a few days of culture, as shown by the gradual decline in *P. murina* gene expression in Fig. 2A. In contrast, the expression of *P. murina* genes in PC organisms cultured in the PCLS model was generally stable throughout the incubation period (Fig. 2B and C), which is consistent with fungal survival. In addition, total RNA-sequencing data of PC-inoculated PCLS on day 3 and day 14 of culture showed that 99% of *P. murina* genes expressed during *in vivo* infection of CD4-depleted C57Bl/6 mice infected with *P. murina* for 2 weeks were maintained in the PCLS model (Fig. S2A and B). When comparing the day 14 PCLS transcriptomic data with the day 3 data, only one *P. murina* transcript was differentially expressed between day 14 and day 3 of PCLS culture (Table S1), which supports that the PC transcriptome was stable in the PCLS culture systems over the study period. Furthermore, *P. murina* mitochondrial transcripts were also stably expressed in the PCLS model (Fig. S3). Immunohistochemistry (IHC) staining, using an anti-*P. murina* polyclonal antibody, of PCLS sections that were cultured with *P. murina* inoculum for 14 days, showed evidence of fungal aggregation and possible biofilm formation (Fig. 2G and H). Furthermore, like in the naïve PCLS model, the PC-inoculated PCLS expressed both type I and type II cell markers throughout the incubation period, suggesting the presence of both cell types in the lung tissues over the study period (Fig. 2D through F). These data indicate that the PCLS system can provide a platform that can sustain the survival and potential growth of *P. murina.*

To further confirm the viability of the cultured organisms, we inoculated naïve immunodeficient, *Rag2^-/-^Il2rγ^-/-^*, mice with aliquots of the day 14 PCLS-cultured *P. murina*, followed by harvesting the lungs, extracting total lung RNA from the right lung by Trizol, and measuring the lung fungal burdens, 4 weeks post inoculation. The lung fungal burdens of the inoculated mice were measured by quantification of the *P. murina* mitochondrial small subunit (mtSSU) ribosomal RNA gene levels in the right lung using RT-qPCR. Our data showed an increase in the levels of the *P. murina* mtSSU rRNA in the lungs of inoculated mice compared to the starting inoculum (Fig. S1B). These data suggest that the cultured PC organisms were still viable, after 14 days of culture, to initiate a productive infection in immunodeficient mice.

### Survival of *P. murina* in immunocompetent PCLS model vs immunodeficient PCLS model

**Fig 3:**
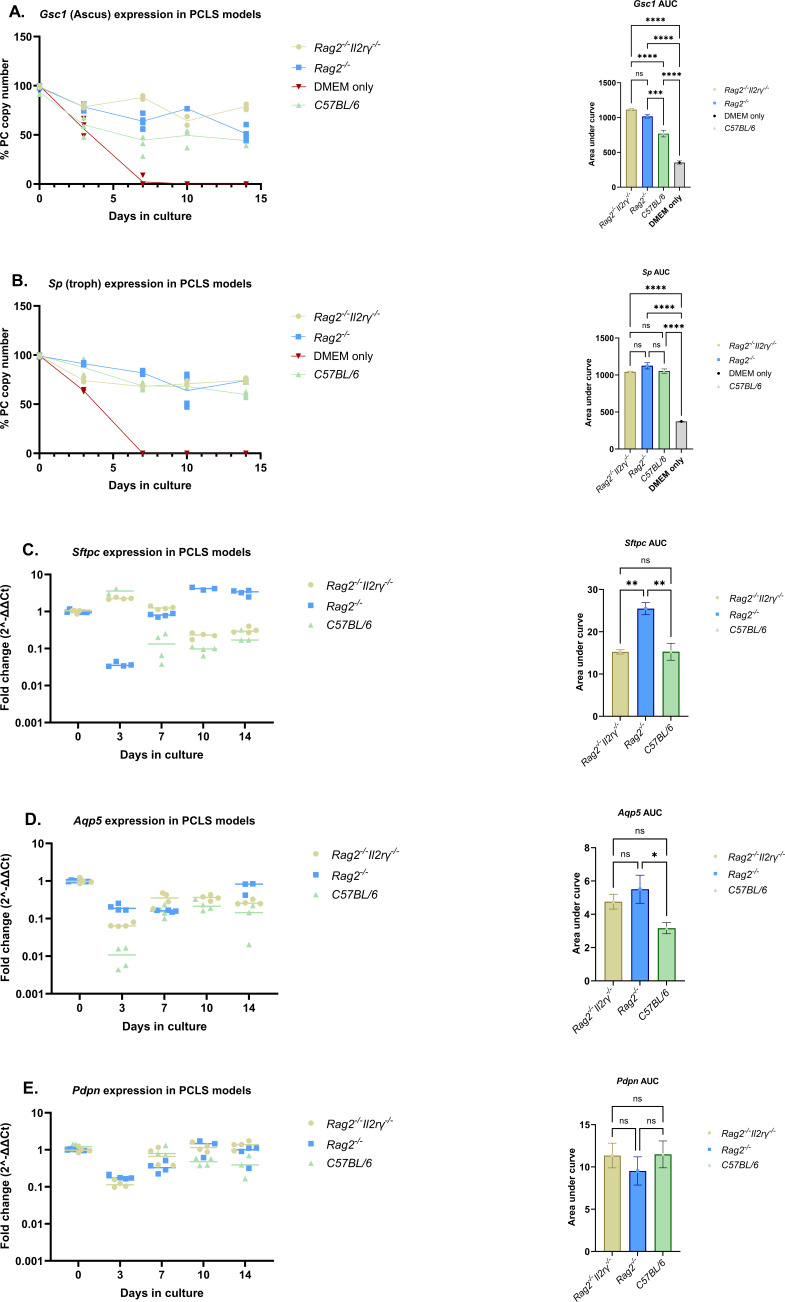
Survival of *P. murina* on PCLS derived from various mice species over time. Expression profiles of the *P. murina* (**A**) ascus (*Gsc1*)- and (**B**) troph (*Sp*)-specific transcript over time in PCLS models of different mice species. (**C–E**) *Sftpc*, *Aqp5*, and *Pdpn* gene expression profiles over time in *P. murina* infected PCLS and the respective AUC. AUC for all gene expression profiles was computed as described in Fig. 1. Statistical comparisons between groups were done by ordinary one-way analysis of variance (ANOVA) with Turkey’s multiple comparisons test with *n* = 4 PCLS per time point for all experiments and *P* designated as ns: *P* ˂ 0.05, * : P ≤ 0.05, ** : P ≤ 0.01, *** : P ≤ 0.001, and **** : P ≤ 0.0001.

Since PC thrives in immunocompromised host lungs, we wanted to understand if immune cells such as lung NK cells or innate lymphoid cells that populate the lung during fetal development may limit fungal growth in the PCLS model. To that end, we evaluated the survival of *P. murina* on PCLS derived from immunocompetent (*C57BL/6*) mice in comparison to PCLS derived from immunodeficient mice (*Rag2* and *Rag2^-/-^Il2rγ^-/-^*). For this study, we cultured *P. murina* inoculum on PCLS derived from *Rag2*, *Rag2^-/-^Il2rγ^-/-^*, and wild type *C57BL/6* mice in air-liquid interface models. Consistent with the data shown in Fig. 2A through C, fungi cultured on PCLS remained viable throughout the study period in contrast to the fungi cultured in DMEM which gradually died within a few days (Fig. 3A and B). However, PCLS models from different mice species supported the survival of *P. murina* to a different degree over the study period, with immunocompromised mice (*Rag2^-/-^* and *Rag2^-/-^Il2rγ^-/-^*) PCLS models performing better compared to the wild type *C57BL/6* mice PCLS model. This was most prominent for the ascus marker, *Gsc1*, which showed more stable expression in PCLS tissues from immunodeficient mice (Fig. 3A). Furthermore, PCLS from the three mice species expressed both type I (*Aqp5* and *Pdpn*) and type II (*Sftpc*) cell markers over time (Fig. 3C through E), which is consistent with viability of both cell types in the respective PCLS models. These data further support the fact that the PCLS system, regardless of the mouse type they are derived from, sustains the viability of *P. murina* longer than culture media alone. Moreover, it suggests that local lung immune cells, such as tissue-resident memory (TRM) cells, lung NK cells, or innate lymphoid cells, may limit fungal growth in the PCLS model.

### Antibiotic susceptibility testing of *P. murina* in PCLS model

**Fig 4:**
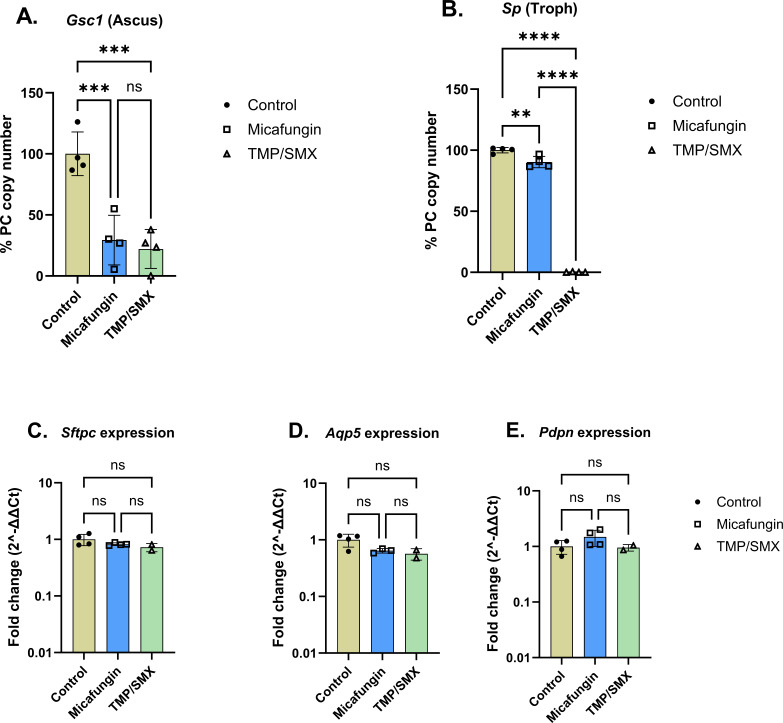
*In vitro* antibiotic targeting of *P. murina* on PCLS model. (**A and B**) Real-time qPCR of troph and ascus transcripts in PCLS models of non-treated (control sample) or micafungin (9 µg/mL)-treated or TMP-SMX (150 µg/mL)-treated samples. *P. murina Gsc1* and *Sp* copies in each group were expressed as a percentage of the copies in the control sample. (**C–E**) Real-time PCR fold changes of host lung genes in control (non-treated), micafungin-, and TMP/SMX-treated PCLS culture models. Data presented are an average of two independent experiments with number of PCLS, *n* = 4, per each time point. Statistical comparisons between groups were done by ordinary one-way ANOVA with Turkey’s multiple comparisons test, with ns: *P* ˂ 0.05, *: P ≤ 0.05, ** : P ≤ 0.01, *** : P ≤ 0.001, and **** : P ≤ 0.0001.

We next tested the effect of the commonly used antifungal drugs, trimethoprim-sulfamethoxazole (TMP-SMX), which has activity against the troph and ascus, and echinocandin and micafungin, which are active against the ascus, in the PCLS model. In this study, *Rag2^-/-^Il2rγ^-/-^* mice-derived PCLS was inoculated with *P. murina* in transwell culture plates, antibiotics were added to the basolateral media starting day 7, and the cultures were harvested on day 14. Subsequently, total RNA was extracted from the cultures using Trizol LS and *P. murina* troph (*Sp*)- and ascus (*Gsc1*)-specific transcript levels as well as lung gene (*Sftpc*, *Aqp5*, *Pdpn*, and *Hprt*) expressions were quantified by RT-qPCR. As anticipated, TMP-SMX had significant activity against both the ascus and troph transcripts, whereas micafungin only showed activity against the ascus (Fig. 4A and B). Furthermore, there was no statistical difference in the expression of the tested lung genes between the control (untreated) and the antibiotic-treated PCLS (Fig. 4C, D and E), which is indicative of the absence of toxicity effects of the antibiotics on the lung cells at the tested concentrations. Thus, these data support that the PCLS model may be useful for *in vitro* anti-fungal studies of PCP.

## DISCUSSION

PCP remains a common infection in immunocompromised hosts as well as a frequent cause of pneumonia in HIV-negative infants (26). Current antibiotic regimens are still largely based on work in the 1970s that antagonizing folate metabolism is therapeutic in active infection (27, 28) and is the most common form of fungal prophylaxis in at-risk individuals (27). Despite this, there are a large number of patients that cannot tolerate TMP-SMX regimens due to toxicity within the hematopoietic compartment or allergy (29–31). Many human pathogen genomes were sequenced as early as the late 1990s due to the ability to culture these organisms. The first fungal genome to be completely sequenced was *Saccharomyces cerevisiae*, reported in 1996 (32). The main reason why it took another 18–20 years for the *P. jirovecii genome* to be sequenced was largely due to the lack of an *in vitro* culture system. The inability to culture the organism has thus hindered elucidation of the fungal genome until recently (33–35) and delayed high-throughput screening to screen large sets of anti-fungal compounds. Furthermore, it has been difficult to study fungal attachment to the type I pneumocyte, which is likely key to the fungal life cycle.

Given the close proximity of the troph to the type I pneumocyte (36), we reasoned that a system that replicates alveolar architecture may permit *in vitro* studies of *Pneumocystis* spp. The PCLS model has preserved alveolar architecture and expresses markers of type I and type II cells, and thus, we hypothesized that this would be a useful model to study critical aspects of PC biology. Previous studies from our lab validated the use of *Gsc1* (ascus-enriched expression marker) and *Sp* (trophozoite-enriched expression marker) as life-form specific makers for PC (25). Furthermore, our lab validated the use of RNA assays as a measure of fungal viability in a prior *in vivo* model that supports fungal replication (25, 37). We thus used a molecular approach to determine the viability of *P. murina* organisms in PCLS culture model by assessing the expression of *Gsc1* (an ascus-specific transcript) and *Sp* (a troph-specific transcript) by real-time qPCR, over time. In this system, we could not use the ATP assay to measure the viability of *P. murina* organisms in culture, as shown in previous studies (38, 39), as the signal would not be fungal specific.

Although we did not detect evidence of growth, in contrast to fungi in media alone, fungi in the PCLS model remained viable for up to 14 days. Molecularly, our data show evidence of survival of both the asci and trophic forms over the study period. The fact that the asci and trophic markers show tremendous stability in the PCLS culture model over time is consistent with fungal survival. Moreover, the fact that the life form specific transcripts were reduced by life form specific anti-fungal compounds (Fig. 4A) supports the viability of the specific life forms in the PCLS culture model. Further confirmation of the viability of PCLS-cultured *P. murina* organisms was done by challenging immunosuppressed mice with PC organisms that have been cultured for 14 days. Our data show that *P. murina* organisms, at day 14 of culture, were still viable to initiate a productive infection in immunodeficiency mice (Fig. S1B). Moreover, IHC staining data (Fig. 2G and H) showed evidence of fungal aggregation and possible biofilm formation in *P*. murina-inoculated PCLS at day 14 of culture. This is consistent with previous findings that *Pneumocystis* spp. form biofilms within the host lung alveoli, and this is one of the proposed mechanisms by which these organisms spread throughout the host lung and cause infection (40). We also observed slightly more stable fungal survival in PCLS from *Rag2^-/-^Il2rγ^-/-^* mice (Fig. 3A), suggesting that cells within *C57BL/6* or Rag*2^-/-^* lungs such as innate lymphoid cells or NK cells may limit fungal growth in this model, which is consistent with the work of Kelly and colleagues (41). Thus, PCLS from *C57BL/6* mice could be used to study how tissue-resident memory cells function in the PCLS model, and PCLS from Rag*2^-/-^* mice could be used to isolate the role of NK cells and innate lymphoid cells.

More importantly, we were able to demonstrate that the PCLS system has value in testing anti-fungal molecules such as TMP-SMX as well as echinocandins. Most current antifungal testing for PC is conducted *in vivo* which requires a large number of animals and their discomfort as well as time to conduct the studies. On the other hand, about up to 20 PCLS can be prepared from a single lobe of the mouse lung (42), and these can be adapted to multi-well assays to allow potential high-throughput screening of novel anti-fungal compounds.

Although we can maintain a uniform size of the PCLS, the cellular composition may vary from slice to slice based on regional variability within the lung (13). This may result in differences in the expression patterns of specific lung genes in different PCLS tissues as observed in all groups (Fig. 1 to 4) of our study. As shown in previous studies, PCLS lose certain cell populations, such as pneumocytes and lymphocyte cells during long-term cultivation (43, 44). Thus, the general decline in the expression of the tested lung genes over time may be due to the decline in the cells that express them. In addition, a recent study by Shiraishi and colleagues showed that the biophysical forces mediated by normal breathing restrict alveolar type I cells from differentiating of into type II cells in an adult lung (45). Due to the lack of these forces in the PCLS system, we speculate that the significant decline in *Aqp5*, an alveolar type I cell marker, in both naïve and PC inoculated *PCLS* (Fig. 1C and 2E) may be partly due to declining type I cells because of differentiation into type II cells, but this is subject to ongoing investigation.

Recent analyses of *Pneumocystis* spp. genomic data (25) show that surface glycoproteins represent the most divergent gene family across *Pneumocystis* species. These differences likely allow differential host attachment. Based on work in both human and rat PCLS models (14–17), we predict that these systems may be useful to study *P. jirovecii* and *carinii*, respectively. In this study, we only assessed the viability of the organisms up to 14 days based on the results from the cell viability assays (Fig. 1A) and findings from previous studies that maintained viability and structural integrity of human and murine PCLS for 15 days (20, 21). We believe that, with optimization and use of advanced culture techniques, the viability and structural integrity of PCLS can be prolonged beyond 2 weeks. Future studies will investigate strategies to optimize and improve the efficiency of the system, morphological changes of the PC organisms in culture over time, mechanisms of fungal attachment to primary cells, as well as various macrophage populations that have fungicidal activity.

### Conclusions

Our data show that PCLS supports the survival of *P. murina* organisms over the 2-week study period. We believe that with further refinement, the PCLS system has the potential to sustain *Pneumocystis* spp. growth. We presume that the lack of PC growth in this system may be due to missing metabolites or micronutrients in the culture media. Hence, we are currently pursuing metabolomic studies in this system compared to *in vivo* infection to see if there are metabolites that are present in the *in vivo* infection that are missing in the PCLS system. This will provide a basis for supplementing the culture media of the PCLS system. Moreover, our data show the potential use of the PCLS system for *in vitro* anti-fungal testing. Most of the current anti-fungal testing is conducted *in vivo* which has the expense of animals, time, as well as the per diems. However, a large number of PCLS can be generated from a single lung tissue, and these can be adapted to multi-well assays to allow high throughput screening of novel anti-fungal compounds. Furthermore, as the PCLS model is also well established in human lungs (16, 20), we believe this could be adapted for *P. jirovecii* (the species that infect humans). Although studies of this system are still on-going, we think it is important to acquaint the scientific community with the potential of the PCLS system as an *ex vivo* platform to study the biology of *Pneumocystis* spp. This will go a long way in the field of PC to address crucial questions about the biology of this fungal pathogen.

## MATERIALS AND METHODS

### Mice

Wild type C57BL/6J mice were obtained from the Jackson Laboratory (Bar Habor, ME, USA). *Rag2^-/-^* and *Rag2^-/-^Il2rγ^-/-^* mice, on a *C57Bl/6* background, were originally obtained from Taconic Biosciences (Troy, New York, USA). Male and female mice between 6 and 10 weeks of age, an average weight of 25 g, were used in this study. All mice were bred and housed in specific pathogen-free rooms within the Tulane University School of Medicine animal facilities, in accordance with the guidelines of the institution.

### Generation of PCLS

PCLS were generated according to the procedure by Klouda and colleagues (18) with a few modifications. Briefly, male and female mice (6–10 weeks old) were euthanized by isoflurane overdose followed by exposing the visceral organs using dissection instruments. Blood was flushed by injecting 10 mL of 1× phosphate-buffered saline (PBS; ThermoFisher Scientific, 70011069) slowly through the right ventricle using a 23G × ¾ (0.6 × 19 mm) PrecisionGlide needle (BD, 305143). The lungs were inflated by injecting 2.5 mL of warm (below 40°C) of 2% low melting point agarose (Invitrogen, 16520100) into the trachea through a cannula connected to a 3 mL syringe. The instilled agarose was solidified by pouring cold 1× PBS over the lungs. After solidification, the lung tissue was separated into lobes using a disposable scalpel and submerged in 1× DMEM (ThermoFisher Scientific, 10569–010) containing 100× penicillin/streptomycin (*P*/S) in a petri dish. The separate lobes were uniformly cut into 300 µm thick slices using a Leica VT1200 Semi-Automatic Vibrating Blade Microtome at a speed of 0.2 mm/s. All slices were collected in chilled 1× PBS before being transferred into 12-well culture plates. The freshly cut slices were placed into sterile submerged well (CELLTREAT, 229111) or transwell (CELLTREAT, 230621) tissue culture plates with 1× DMEM containing 1× P/S and incubated in 5% CO_2_ at 37°C overnight to allow recovery from slicing. The viability of the lung slices was tested using PrestoBlue cell viability reagent (ThermoFisher Scientific, A13261) as outlined below.

### Viability of PCLS

The viability of PCLS was assessed using the PrestoBlue cell viability assays (ThermoFisher Scientific, A13261) according to the manufacturer’s protocol. Briefly, PCLS (three slices per well) was placed in a 12-well culture plate containing 450 µL of DMEM and 50 µL PrestoBlue cell viability reagent per well, before incubating for 1 hour at 37^o^C. Heat-killed PCLS was used as a negative control. Following the 1-hour incubation, the supernatant was harvested, and fluorescence of the supernatant was quantified at 560/590 nm (excitation/emission) using a BioTek Synergy H1 Multimode microplate reader (Agilent Technologies, Inc., Santa Clara, CA, USA). The fluorescent intensities of the test PCLS were normalized to the negative control (heat-killed PCLS). The supernatant from the PCLS was replaced by fresh DMEM, and the slices were maintained in culture at 37°C and 5% CO_2_. The viability assays were repeated up to 17 days of culture.

### Preparation of *P. murina* inoculum

*P. murina* organisms were isolated from infected Rag2^-/-^Il2rγ^-/-^ whole lungs as described by Dai and team (46) with few alterations. Briefly, PCP infected whole lungs stored in 1 mL sterile Dulbecco’s PBS at −80°C were thawed and strained through a 70 µm filter. The resulting lung suspension was then centrifuged at 1,500 × *g* for 10 minutes at 4°C. The pellet was resuspended in 1 mL of 1× PBS, and the asci were counted microscopically. Subsequently, the concentration of the inoculum was adjusted to approximately 1 × 10^6^ asci per mL. For all experiments, the starting inoculum was denoted as day 0.

### PCLS culture

Following incubation at 37°C, 5% CO2 overnight, PCLS were moved to new 12-well tissue culture plate (CELLTREAT, 229112) with 450 µL fresh 1× DMEM containing 1× P/S, or permeable cell culture inserts, pore size 4 µm, packed in 12-well plate (CELLTREAT, 230621) with 450 µL fresh 1× DMEM containing 1× P/S. Subsequently, 120 µL (1 × 10^6^ asci per mL) of the *P. murina* inoculum was added to the submerged well or transwell tissue culture plate containing the PCLS and incubated in 5% CO_2_ at 37°C for 2 weeks. Total RNA was isolated from the PCLS cultures at days 0, 3, 7, 10, and 14 using TRIzol LS reagent as described below.

### Antibiotic susceptibility tests

PCLS was placed in sterile permeable cell culture inserts (pore size, 4 µm) packed in 12-well plate containing 450 µL fresh 1× DMEM with 1× P/S following incubation in 5% CO_2_ at 37°C overnight. The PCLS were inoculated with 120 µL (1 × 10^6^ asci per mL) of *P. murina* inoculum per well and placed back into 5% CO_2_ and incubated at 37°C for 7 days to allow for acclimatization. At day 7, the media was removed from the culture plates and exchanged with fresh 1× DMEM alone in control samples; or 1× DMEM containing either 9 µg/mL micafungin (47) or 150 µg/mL (SMX component) TMP-SMX (48) in the experimental samples. The plates incubated for another 7 days in 5% at 37°C. The cultures were harvested at day 14, and total RNA was isolated from the control and experimental samples using trizol LS. The effects of the antibiotics on *P. murina* were determined by measuring of *P. murina* troph (*Sp*)- and ascus (*Gsc1*)-specific transcript levels by RT-qPCR.

### RNA isolation and RT-qPCR quantification

PCLS cultures with or without *P. murina* were harvested in TRIzol LS reagent (ThermoFisher Scientific, 10296010) and homogenized using a PRO Scientific Bio-Gen PRO200 Homogenizer (01–4607). This was followed by the isolation of total RNA in accordance with the TRIzol LS Reagent manufacturer’s protocol. Subsequently, cDNA was synthesized from 150 ng of total RNA per 20 µL reaction using the Bio-Rad iScript cDNA Synthesis Kit (1708841). The expression of host lung genes (*Sftpc, Aqp5* and *Pdpn*) was quantified by RT-qPCR using SsoAdvanced Universal SYB Green Supermix (Bio-Rad, 1725270), TaqMan Gene expression assays mix (ThermoFisher Scientific; Mm00488146_g1, or Mm00437578_m1, or Mm00494716_m1) and 2 µL of cDNA per 20 µL reaction. All host lung gene expressions were normalized using the mouse *Hprt* gene expression assay (ThermoFisher Scientific, Mm03024075_m1). Expression of *P. murina* genes, *Gsc1*, for the ascus, and *Sp*, for the troph, was determined by RT-qPCR using SsoAdvanced Universal SYB Green Supermix (Bio-Rad, 1725270), IDT (Coralville, Iowa, USA) custom primers, and 2 µL of cDNA per 20 µL reaction. The *Gsc1* forward and reverse primers were 5′-ATT ATG CGC CGG AAT ATG G-3′ and 5′-ACT GAA GAG GAC GCT GAT-3′ respectively. The *Sp* forward and reverse primers were 5′-AGT AGG TGT CTC GTC ACA TAA AG-3′ and 5′-RCT GGA AGG GTT GAG TAT CAT AGA G-3′, respectively. RNAseq methods are in the supplemental information. The RNAseq data have been deposited in GEO, accession number GSE247615.

### Preparation of tissues for paraffin-embedded sections and H&E staining

PCLSs for paraffin embedding and H&E staining were fixed 4% methanol free formaldehyde solution (ThermoFisher Scientific, 28908) in 1× PBS over night at 4°C. The preparation of unstained paraffin-embedded sections as well as H&E-stained slides was done at the Tulane School of Medicine Histology Research Core Laboratory.

### IHC staining

IHC staining of the paraffin-embedded sections was done using the Anti-Ig horeseradish peroxidase (HRP) Detection kit (BD,551011), and polyclonal mouse sera was obtained from a *Rag2^-/-^Il2rγ^-/-^* mouse that has been infected with *P. murina* for 8 weeks. IHC staining was done in accordance with the Anti-Ig HRP Detection kit manufacturer’s manual. Briefly, unstained paraffin-embedded slides were deparaffinized and rehydrated as described in the instruction manual. Polyclonal mouse serum that has been diluted in an antibody diluent (1:500), provided in the kit, was applied to the slides and incubated overnight at 4°C. A biotinylated anti-Ig secondary antibody was applied to the slides following three washes in PBS and incubated for 30 minutes at room temperature. This was followed by three more washes in PBS, the addition of Streptavidin-HRP to the tissues on the slides, and incubation for 30 minutes at room temperature. The slides were then rinsed three times in PBS, and diaminobenzidine (DAB) substrate solution was added and allowed to incubate for 5 minutes. This was followed by three washes in PBS and counterstaining using modified Mayer’s Hematoxylin (Epredia, 72804) for 60 seconds. The slides were then rinsed thoroughly in water, dehydrated, and coverslip according to the instruction manual. Imaging of the immunochemical- and H&E-stained slides was done using a brightfield microscope (Olympus BX53, 1E39225).

### Statistics

Graphs for the data were generated and analyzed statistically using the GraphPad Prism software, version 9.5.1. The number of lung slices (PCLS) examined in each experiment is defined as “*n.*” Data presented are an average of two independent experiments with number of PCLS, *n* = 4, for each time point. Area under the gene expression curve was calculated by considering the area under each gene expression profile between day 0 and day 14, using the GraphPad Prism software. Expression of the *P. murina* genes (*Gsc1* and *Sp*) over time was presented as a percentage of *P. murina* ascus or troph copies in the starting inoculum (day 0). Statistical comparisons between two groups were done using a simple two-tailed unpaired Student’s *t* test, while statistical comparisons between three or more groups were done by ordinary one-way ANOVA with Turkey’s multiple comparisons test. Values were presented as mean ± SEM, and *P* values were denoted as follows: ns: *P ˂* 0.05, *: P ≤ 0.05, ** : P ≤ 0.01, *** : P ≤ 0.001, and **** : P ≤ 0.0001.

## SUPPLEMENTAL MATERIAL

File S1 mbio.01464-23-s0001.csv *P. murina* transcript abundance day 3 PCLS.

File S2 mbio.01464-23-s0002.csv *P. murina* transcript abundance day 14 PCLS.

File S3 mbio.01464-23-s0003.csv *P. murina* transcript abundance day 14 *in vivo*.

Supplemental Material mbio.01464-23-s0004.pdf Supplemental methods and data.

ASM does not own the copyrights to Supplemental Material that may be linked to, or accessed through, an article. The authors have granted ASM a non-exclusive, world-wide license to publish the Supplemental Material files. Please contact the corresponding author directly for reuse.
