# Supplementary material for: Precision-cut lung slices as an ex vivo model to study Pneumocystis murina survival and antimicrobial susceptibility
Source: mBio. 2023 Dec 20;15(1):e01464-23. doi: 10.1128/mbio.01464-23 (PMC10790776; doi:10.1128/mbio.01464-23)
Supplement: Supplemental Material — Supplemental methods and data. [file mbio.01464-23-s0004.pdf]

Supplementary Information

Materials and methods

Inoculation of immunosuppressed, *Rag2$^{-/-}$Il2rγ$^{-/-}$*, mice with cultured *P. murina* and quantification of fungal lung burdens.

A total of 8 aliquots (120 µL each) of *Pneumocystis murina* inoculum (1X10^5 asci per mL) were cultured separately in air-liquid interface PCLS system as previously described under "PCLS culture". The cultures were harvested on day 14 of culture and *P. murina* organisms were isolated from the mixed cultures by centrifugation at 1500*xg* for 10 minutes at 4°C. The resulting pellet was washed twice in 1 mL of 1x PBS by differential centrifugation and resuspended in 1 mL 1x PBS. The number of *P. murina* mitochondrial small subunit (mtSSU) ribosomal RNA copies per mL of suspension was approximately 4 x 10^3 by RT-qPCR. Four 6-weeks old male and female naïve *Rag2$^{-/-}$Il2rγ$^{-/-}$* mice were challenged with 100 µL of the prepared inoculum via oral pharyngeal instillation as previously described [10, 43]. The mice were sacrificed four weeks post inoculation and total RNA was extracted from the homogenized right lung using Trizol reagent (ThermoFisher, 15596018) according to manufacturer's protocol. Subsequently, cDNA was synthesized from 1 µg [10, 44] of total RNA per 20µL reaction using the Bio-Rad iScript cDNA Synthesis Kit (1708841). The lung *P. murina* burdens were subsequently quantified by real-time PCR using SsoAdvanced Universal Probes Super mix (BIO-RAD, 1725281), with the following *P. murina*-specific **mitochondrial small subunit (mtSSU) rRNA primers**: Forward: 5′- 5′-TCGGACTTGGATCTTTGCTTCCCA; Reverse: 5′-CATTCCGAGAACGAACGCAATCCT; and FAM probe: 5′-TCATGACCCTTATGGAGTGGGCTACA (all from IDT). Threshold cycle values were converted to RNA copy numbers using a premade standard of known Pneumocystis mtSSU rRNA as described previously standards were prepared as described previously [36].

RNA sequencing:

RNA extraction and quantification

Total RNA was extracted from *P. murina* inoculated PCLS on day 3 and day 14 of culture using TRIzol LS reagent (ThermoFisher Scientific, 10296010) in accordance with the TRIzol LS Reagent manufacturer's protocol. The total RNA was DNase treated and quantified using the Qubit RNA HS assay kit (Thermo Fisher Scientific, Q32855). RNA quality was determined on an Agilent TapeStation 4150 using an Agilent RNA ScreenTape (Agilent, 5067-5576).

NEB total RNA Library Prep

About 10 ng of each total RNA sample was used to generate total RNA libraries using the NEBNext rRNA Depletion Kit v2 (Human/Mouse/rat) (NEB #E7400) and NEBNext UltraII Direction RNA Library Prep Kit for Illumina (NEB #7760). Subsequently, Final cDNA libraries containing NEB Dual Index (#E6440) were quantified using Qubit dsDNA HS assay kit (Thermo Fisher Scientific, Q32854). The quality of the libraries was determined by running each on an Agilent TapeStation 4150 using an Agilent D1000 ScreenTape (Agilent: 5067-5582). Smear

analysis was performed using Agilent TapeStation Software (Version 4.1.1) with a range of 200-800bp to determine the average size of each library. The molarity of each library was then calculated based on size and concentration. All libraries were pooled at final concentration of 750pM with a spike-in of 2% PhiX control library v3 (Illumina, FC-110-3001). A mixture of pooled libraries was loaded on an Illumina NextSeq P2 (100) reagent cartridge (Illumina, 20046811). Single read and dual indexing sequence, 100 x 8 x 8, was performed on NextSeq2000, yielding approximately 66M Single reads per sample. Fastqs generated by Illumina BaseSpace DRAGEN Analysis Software (Version 1.2.1) were applied for further data analyses. The data generated was compared to RNA sequencing data from an *in-vivo P. murina* infection study.

Results

Verification of viability of the cultured *P. murina* organisms

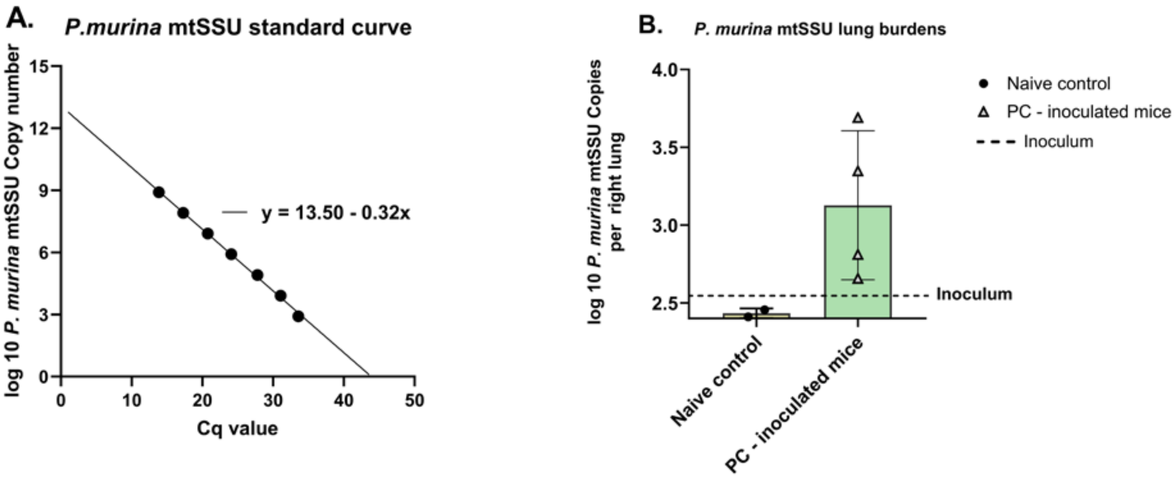

Fig S1 Lung fungal burdens of *Rag2*$^{-/-}$*Il2rγ*$^{-/-}$ inoculated with *P. murina* cultured in the PCLS system for 14 days. **(A)** *P. murina* mtSSU rRNA copies standard curve. **(B)** *P. murina* mtSSU rRNA lung burdens of mice inoculated with cultured *P. murina* organisms. Each data point represents a mouse.

Table S1 Table of differentially expressed *P. murina* genes in *P. murina* inoculated PCLS at day 3 and day 14 of culture, from Cuffdiff.

Gene I.D	Gene symbol	Description	Abundance on Day 3 of culture	Abundance on Day 14 of culture	Day3 vs Day14 fold change	Day 3 vs Day 14 P value
19894971	PNEG_01274	BRO1-domain-containing protein *C	29.61750488	0	0	0.031364

Relative abundance of *Pneumocystis* genes in *P. murina* inoculated PCLS vs in-vivo infection by RNA sequencing.

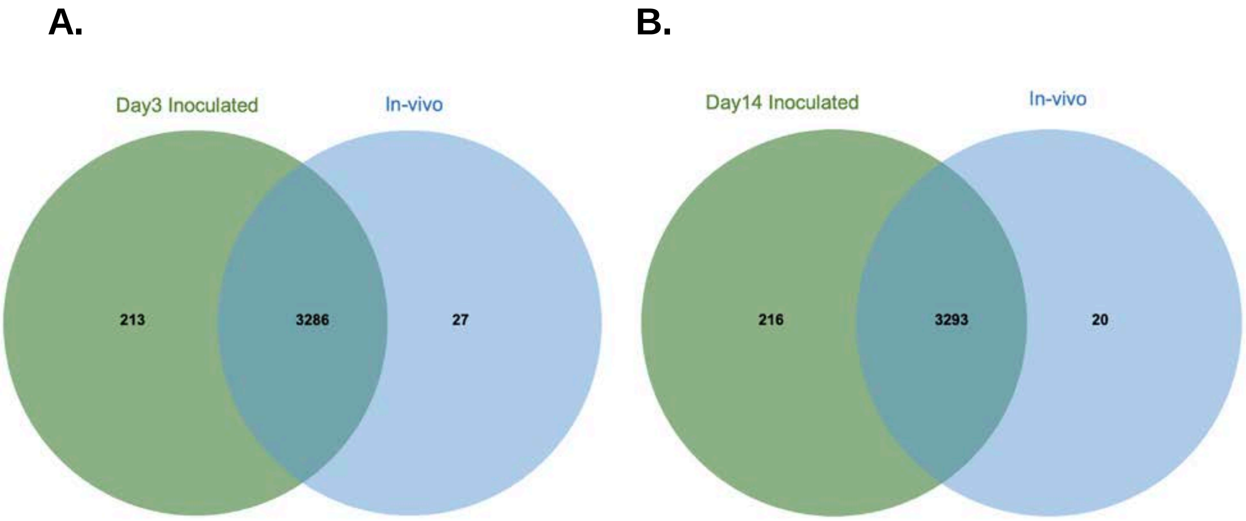

Fig S2 RNA sequencing data Venn diagrams comparing the expression of *Pneumocystis murina* genes in PC-inoculated PCLS at day 3 and day 14 of culture with 14 day *in-vivo P. murina* infection study (GK1.5 treated/CD4 depleted mice). **(A, B)** Venn diagrams showing the common genes between **(A)** PC-inoculated PCLS at day 3 of culture and the 14 day *in-vivo* infection study, and **(B)** PC-inoculated PCLS at day 14 of culture and the *in-vivo* infection study. To generate this Venn diagram, day 3 inoculated and day 14 inoculated abundance were used from day 3 and day 14 of culture versus Naïve PCLS comparisons. For the *in-vivo* study, GK 1.5 abundance was used. Abundance threshold was set at >5 for all data curations.

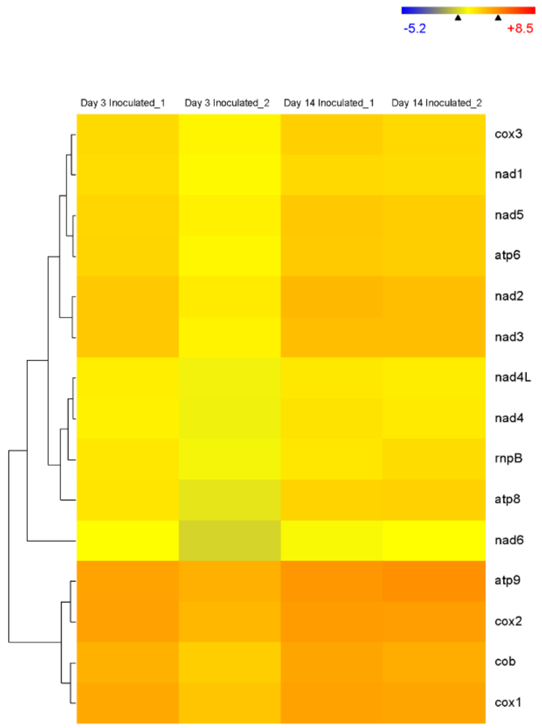

Fig S3 Heatmap of *Pneumocystis murina* mitochondrial OXPHOS genes measured by linear weighted RPKM-CN normalization in *P. murina* inoculated PCLS on day 3 and day 14 of culture.

Supplemental CSV Files

1. Day 3 *P. murina* transcript abundance in the PCLS model: This table contains *P. murina* transcript abundance (FPKM) at day 3 of PCLS culture.

2. Day 14 *P. murina* transcript abundance in the PCLS model: This table contains *P. murina* transcript abundance (FPKM) at day 14 of PCLS culture.

3. Day 14 P. murina transcript abundance CD4 depleted mouse lung model. This table contains *P. murina* transcript abundance (FPKM) at day 14 after in vivo propagation in CD4 dependent (via GK1.5 monoclonal antibody administration) C57Bl/6 mice.
